# Risk prediction for severe disease and better diagnostic accuracy in early dengue infection; the Colombo dengue study

**DOI:** 10.1186/s12879-019-4304-9

**Published:** 2019-08-01

**Authors:** Ponsuge Chathurani Sigera, Ranmalee Amarasekara, Chaturaka Rodrigo, Senaka Rajapakse, Praveen Weeratunga, Nipun Lakshita De Silva, Chun Hong Huang, Malaya K. Sahoo, Benjamin A. Pinsky, Dylan R. Pillai, Hasitha A. Tissera, Saroj Jayasinghe, Shiroma Handunnetti, Sumadhya D. Fernando

**Affiliations:** 1 Department of Parasitology, Faculty of Medicine, University of Colombo, Colombo, Sri Lanka; 2 Department of Microbiology, Immunology, and Infectious Diseases, University of Calgary, Calgary, Canada; 3 Department of Pathology, School of Medical Sciences, UNSW Sydney, Kensington, Australia; 4 Department of Clinical Medicine, Faculty of Medicine, University of Colombo, Colombo, Sri Lanka; 5 Department of Clinical Medicine, Faculty of Medicine, General Sir John Kotelawala Defence University, Colombo, Sri Lanka; 6 Department of Pathology, Stanford University School of Medicine, Standford, USA; 7 Department of Medicine, Division of Infectious Diseases and Geographic Medicine, Stanford University School of Medicine, Standford, USA; 8 National Dengue Control Unit, Colombo, Sri Lanka; 9 The Institute of Biochemistry, Molecular Biology and Biotechnology, Colombo, Sri Lanka

**Keywords:** Dengue, Severe dengue, Dengue virus, Dengue diagnostics, RT-qPCR, LAMP, NS1

## Abstract

**Background:**

A major challenge in dengue management in resource limited settings is the confirmation of diagnosis. Clinical features of dengue often overlap with other infections and molecular diagnostic tools are not readily accessible to clinicians at hospitals. In addition, the prediction of plasma leakage in dengue is also difficult. Hematocrit level and ultrasound scans (combined with clinical parameters) are helpful to detect plasma leakage once it has happened, not before.

**Methods:**

Colombo Dengue Study (CDS) is a prospective cohort study of clinically suspected adult dengue patients recruited from the National hospital of Sri Lanka (within the first 3 days of fever) that aimed to a) identify clinical and basic laboratory test parameters to differentiate dengue from non-dengue fever, b) evaluate the comparative efficacy of loop-mediated isothermal amplification (LAMP) for dengue diagnosis (vs. NS1 antigen test and RT-qPCR) and c) identify early associations that are predictive of plasma leakage or severe dengue. The basic laboratory tests considered here included hematological parameters, serum biochemistry and inflammatory markers.

**Results:**

Only 70% of clinically suspected patients were confirmed as having dengue by either the NS1 antigen test or RT-qPCR. On a Bayesian latent class model which assumes no “gold standard”, LAMP performed equally or better than RT-qPCR and NS1 antigen test respectively. When confirmed dengue patients were compared with others, the earlier group had significantly lower lymphocyte counts and higher aspartate aminotransferase levels (AST) within the first 3 days of fever. Confirmed dengue patients with plasma leakage had a lower mean age and a higher median baseline AST level compared to those without plasma leakage (*p* < 0.05).

**Conclusion:**

Clinical suspicion overestimates the true number of dengue patients. RT-LAMP is a potentially useful low-cost diagnostic tool for dengue diagnosis. Confirmed dengue patients had significantly higher AST levels and lower lymphocyte counts in early disease compared to others. In confirmed dengue patients, younger age and a higher AST level in early infection were associated with subsequent plasma leakage.

**Electronic supplementary material:**

The online version of this article (10.1186/s12879-019-4304-9) contains supplementary material, which is available to authorized users.

## Background

Dengue, a mosquito borne viral infection has become a significant public health problem with an estimated 3.9 billion people in 128 countries being at risk of infection [1, 2]. Each year, up to 400 million people are infected and 22,000 die from severe dengue globally [3]. In endemic tropical countries, seasonal epidemics can affect large numbers of patients over a short period of time, overwhelming the available healthcare resources. Most dengue related deaths are preventable [4].

Sri Lanka, a tropical island in the Indian Ocean with a population of 21 million people, suffered its largest dengue epidemic in 2017, with 186,101 cases and over 300 dengue related deaths [5]. A significant challenge in management is the confirmation of diagnosis as the clinical features overlap with other prevalent infections such as flu and leptospirosis [6]. The commonly used NS1 (Non-Structural protein 1) viral antigen testing (either by rapid testing or ELISA) is most sensitive (55–66%) in the first 3 days of the illness with specificity varying between 89 and 92% [2]. Diagnosis by amplification of viral genomic material (Reverse Transcription and quantitative Polymerase Chain Reaction: RT-qPCR) is mostly restricted to research purposes given the cost. Diagnosis by anti-dengue antibodies is not clinically useful as IgM antibodies appear later in the course of infection and a four-fold rise in IgG only allows retrospective confirmation [7]. Hence, clinicians mostly depend on a clinical diagnosis guided by the serial changes in the hematological parameters. Reverse transcription and Loop-mediated isothermal amplification (RT-LAMP) is a potential alternative diagnostic method for dengue which has proven its cost effectiveness in malaria diagnosis [8–11].

Another challenge in dengue management is the early identification of patients at risk of complications. Patients developing complications have the common underlying feature of increased capillary permeability leading to plasma leakage into the interstitial space. This typically occurs in days 5–7 of fever and lasts for 48–72 h (“critical phase”) [12]. Correct fluid management during this phase is essential to avoid complications. In resource limited clinical settings, the onset of critical phase is estimated by observing serial changes in hematocrit [13]. In more resourceful larger hospitals, serial ultrasound scans are used to detect fluid leakage into the body cavities. Unfortunately, both of these measures detect plasma leakage once it has happened, not before.

We completed a prospective cohort study of patients with clinically suspected dengue fever with the objectives of a) assessing the comparative usefulness of LAMP for confirmation of diagnosis, b) evaluating if early clinical or laboratory investigations could differentiate dengue fever from non-dengue fever and c) if the same can predict the risk of plasma leakage or severe dengue in those with confirmed dengue fever.

## Methods

### Study setting and data collection

The Colombo Dengue Study (CDS) is a prospective observational cohort study conducted by the Faculty of Medicine, University of Colombo at the National Hospital of Sri Lanka (NHSL) which is the largest public sector, tertiary care referral center in the country. This study recruits all non-pediatric (> 12 years of age), non-pregnant patients with an acute febrile illness (≤ 3 days) when two independent medical doctors clinically suspect dengue fever during evaluation. Patients with confirmed plasma leakage or complications of dengue on admission were excluded. Out of the six general medical units in the hospital, three participated in this study (each with a male and female ward). Patients are admitted to all medical units in the hospital after a preliminary assessment by an attending doctor in the out-patients department on an unbiased rotation where each block of six consecutive patients were allocated in order to each of the medical units. This paper considers patients enrolled into the study from October 2017 to May 2018. The calculated sample size (power: 0.85, effect size: 0.5) was 122 [14].

All eligible consenting patients were interviewed by the same investigator using a pre-validated questionnaire. The initial interview collected data on clinical symptoms and patient demography as reported by the patient on admission. Subsequently, patients were seen daily by the same investigator and clinical signs, symptoms and laboratory investigation results were extracted from medical records and by daily patient interviews. Ten clinical signs and symptoms were assessed during the first 3 days of fever in each patient (Additional file 1: Table S1). The basic laboratory parameters assessed within the first 3 days included; hemoglobin level, hematocrit, total leukocyte count and differential leukocyte count, platelet count, C - reactive protein, serum electrolytes, serum creatinine, serum bilirubin and the liver enzyme levels (alanine aminotransferase and aspartate aminotransferase). When there were multiple reports for the same parameter, the earliest report was considered. In addition, the patient’s body mass index (BMI) was also recorded. Patients with a confirmed alternative diagnosis during the hospital stay were excluded and only patients with a confirmed or suspected diagnosis of dengue at discharge remained in the cohort. The management of patients was entirely at the discretion of the physicians of the wards who were not part of the study. However, patient management in all units were standardized according to the national guidelines on dengue management [13].

### Confirmation of diagnosis

Approximately 5–6 ml of blood were collected aseptically to EDTA containers and plasma was separated within 2 h and stored at − 80 °C in 2–3, 1 ml aliquots, one of which was transported to the laboratory at Stanford University, USA for RT-qPCR. NS1 test and the RT-LAMP test were performed at the laboratory at Department of Parasitology, Faculty of Medicine, University of Colombo, Sri Lanka.

In this study, dengue was diagnosed by NS1 antigen testing on admission (one step SD Bioline dengue NS 1 antigen test, Alere SD, USA, 88.65% sensitivity and 98.75% specificity up to day 9 of fever) [15] and by RT-qPCR as described by Waggoner et al. previously [16] with a Ct value cut-off of 38.5 [17]. RT-qPCR also enabled serotyping but as it was done by batch processing for cost-effectiveness, in most cases the diagnosis was made retrospectively after the patient was discharged. For RT-LAMP assay, genomic viral RNA was extracted from 140ul of patient plasma samples using QIAamp RNA mini kit (Qiagen, Germany) and the assay was performed in duplicates using Loopamp RNA amplification kit (Eiken Chemical Co. Ltd., Japan) with the primers previously published by Lau et al. [18] using 1ul of template RNA in each reaction mix [18]. Full description of assay methods is given in Additional file 2.

### Measures of disease outcome

The outcomes assessed in this study were plasma leakage and severe dengue. Plasma leakage was identified according to the National Guidelines on dengue management [13]. In brief, bed-side hematocrit assessments were done at intervals of 6 to 8 h to demonstrate a rise of 20% from baseline. Alternatively, ultrasound evidence (pleural effusions, peri-cholecystic fluid or ascites) was also confirmatory for plasma leakage. Platelet count was used as a surrogate marker to guide the timing of ultrasound examinations as a drop-in platelet count below 100,000/mm3 is observed to precede plasma leakage by 24–48 h [13]. Patients within this time window were examined by ultrasound scans twice daily. Severe dengue was defined according the previously published criteria by World Health Organization (WHO). This included patients with plasma leakage plus shock or fluid overload, severe bleeding or severe organ impairment [19].

### Data analysis

STATA version 13.1 and SPSS (version 24, IBM, USA) were used for the analysis. Descriptive statistics were summarized as measures of central tendency (mean, median) and dispersion (standard deviation and inter-quartile range). Statistical associations for proportional data with dichotomous outcomes were tested using chi-squared test. For continuous data, Shapiro-Wilk test was used to assess the normality of data and then appropriate parametric (independent samples T test) or non-parametric tests (Mann-Whitney rank sum test) were used. Statistical significance was set at *p* < 0.05 with appropriate adjustments for multiple comparisons with a Bonferroni correction. Diagnostic tests were compared using two methods; a) with RT-PCR as the gold standard comparator and b) Bayesian latent class model (BLCM) analysis which assumes no gold standard. Sensitivity, specificity, positive predictive (PPV), negative predictive value (NPV) for each diagnostic test was calculated [20].

## Results

### Descriptive statistics

A total of 122 eligible patients were recruited consecutively. Of these, 86 (70%) tested positive for dengue by either the NS1 antigen testing (55, 63.9%) or RT-qPCR (80, 93.0%). In this study, if patients were positive by either of the tests, it was counted as a confirmed case since the clinical picture was suggestive of dengue according to treating physicians’ clinical judgment. The confirmed dengue patients were predominantly a group of young to middle aged (median age: 27.5 IQR: 20–40 years) males (57/86, 66.3%) resident in the Colombo district where the NHSL is located (Table 1). Of qPCR positive patients, a majority were of DENV-2 serotype (70/80, 87.5%), followed by DENV-1 (4/80, 5%), DENV-3 and 4 (3/80 each, 3.75%). Given the small number of cases from other serotypes, for subsequent comparisons the samples were categorized as DENV-2 and non DENV-2. The number of patients presenting with each of the ten clinical symptoms and signs evaluated is provided in Additional file 1: Table S1 while the mean/median values of first set of routine laboratory investigations is outlined in Additional file 1: Table S2.

**Table 1:** Descriptive statistics of the demography of recruited dengue patients

	Number (*n* – 86)	%	Median + Q1-Q3
Age	–		27.5 years (20–40)
Body mass index	–		20.98 kg/m^2^(18.89–24.51)
*Gender*			
Female	29	33.7	
Male	57	66.3	
*Province of residence*			
Colombo	78	90.70	
Gampaha	6	6.98	
Kaluthara	1	1.16	
Others	1	1.16	
*Level of education*			
No schooling or primary education only	12	13.95	
Grade 6–10	12	13.95	
Passed GCE O/L or equivalent	26	30.23	
Passed GCE A/L or equivalent	18	20.93	
Higher education	11	12.79	
Studying	7	8.14	
*Level of income*			
No income	24	27.91	
Less than Rs 20,000	9	10.47	
Between Rs 20000–30000	13	15.12	
Between Rs 30000–40000	20	23.26	
Between Rs 40000–50000	12	13.95	
More than Rs 50000	8	9.30	
*Preexisting cor-morbidity*			
Diabetic Mellitus	4	4.65	
Hypertension	8	9.30	
Hyperlipidemia	4	4.65	
IHD	3	3.49	
Smoking	15	17.44	
Alcohol consumption	10	11.63	

Twenty-two patients (22/86, 25.58%) had plasma leakage. Of these, ten had severe dengue. This included 8 patients with compensated shock and 4 patients with uncompensated shock. In two patients, compensated shock progressed to uncompensated shock.

### Distinction of dengue vs. non-dengue febrile illness

There were no statistically significant differences regarding the early clinical features between confirmed dengue patients and others. However, following laboratory investigations within the first 3 days of fever showed significant differences between these two groups; The total leukocyte count, neutrophil and lymphocyte counts were significantly lower and the median Aspartate Aminotransferase (AST) level was significantly higher in confirmed dengue patients (Table 2, *p* < 0.05). The association for lymphocyte counts and AST level persisted when all three parameters (including neutrophil count) were combined in a logistic regression analysis (Table 3).

**Table 2:** Early laboratory parameters of confirmed dengue patients and patients who treated as dengue but never confirmed

Laboratory parameter (median)	Confirmed dengue (*n*-86)	Treated as dengue but never confirmed (*n*-36)	*p* value
Hemoglobin (g/dL)	13.65	11.35	0.006
Hematocrit (%)	39.45	33.8	0.025
Platelet count * 10^3^/uL	126	147	0.049
Total leukocyte count * 10^3^/uL	4.12	6.54	0.000*
Neutrophil count * 10^3^/uL	2.39	3.78	0.001*
Lymphocyte count * 10^3^/uL	0.7	1.2	0.003*
AST u/L	50	27	0.000*
ALT u/L	35.5	25.5	0.005
Serum Sodium mmol/L	136	134	0.152
Serum Potassium mmol/L	3.7	3.8	0.333
Serum creatinine umol/L	78	73.5	0.299
C-reactive protein mg/l	16	6	0.045
Serum total bilirubin umol/L	11.6	12.8	0.882

* Statistically significant with the Bonferroni adjusted *p* value of < 0.004

**Table 3:** Association for lymphocyte, neutrophil and AST in dengue confirmation based on NS1 and RT-PCR tests

*Laboratory parameter*	*B*	*S.E.*	*Sig.*
Lymphocytes count	−0.917	0.384	0.017*
AST level	0.027	0.010	0.008**
Neutrophil count	−0.149	0.078	0.057
Constant	1.203	0.659	0.068

*RT-PCR* Reverse transcription and polymerase chain reaction, *NS1* NS1 antigen test, *AST* Aspartate aminotransferase

** *p* > 0.01,*p > 0.05

### Using LAMP for dengue diagnosis

Sixty-one patients were positive for dengue by LAMP (61/122, 50%). Compared to the gold standard RT-qPCR, the sensitivity, specificity, positive predictive value (PPV) and negative predictive value (NPV) of LAMP were 73.8, 95.2, 96.7 and 65.6% respectively. These values were superior to that of NS1 antigen test which is the standard bedside diagnostic test commonly used (Table 4). Interestingly, out of the six dengue patients that were negative by RT-qPCR (but positive for the NS1 test), two were positive by LAMP. On BLCM, LAMP had similar sensitivity [93.4% (80.9–99.9%) vs. 96.6% (90.1–99.7%)] and better specificity compared to RT-qPCR [99.2% (91.7–100%) vs. 69.8% (55.2–82.5%)] (Table 4). LAMP also performed better than NS1 antigen test on both sensitivity and specificity (Table 4).

**Table 4:** Prevalence, sensitivities and specificities, PPV and NPV in confirmation of dengue infection

Parameters	RT-PCR^a^ test was assumed as a perfect gold standard (%)	Bayesian latest class model (%)
Prevalence	65.6 (56.4–73.8)	53.0 (43.0–62.9)
RT PCR^a^		
Sensitivity	–	96.6 (90.1–99.7)
Specificity	–	69.8 (55.2–82.5)
PPV	–	78.4 (66.3–88.5)
NPV	–	94.8 (84.7–99.5)
RT-LAMP^a^		
Sensitivity	73.8 (62.5–82.7)	93.4 (80.9–99.9)
Specificity	95.2 (82.6–99.2)	99.2 (91.7–100)
PPV	96.7 (87.6–99.4)	99.3 (92.3–100)
NPV	65.6 (52.2–77.0)	93.1 (78.2–99.9)
NS 1 test		
Sensitivity	61.3 (49.7–71.7)	75.1 (63.2–84.9)
Specificity	85.7 (70.8–94.1)	89.0 (77.3–96.9)
PPV	89.1 (77.1–95.5)	88.5 (75.3–97.0)
NPV	53.7 (41.2–65.8)	76.0 (63.5–85.8)

^a^*RT-PCR* Reverse transcription and polymerase chain reaction, *NS1* NS1 antigen test, *RT-LAMP* Reverse transcription loop-mediated isothermal amplification, *PPV* Positive predictive value, *NPV* Negative predictive value

### Predictors of plasma leakage and severe dengue

The mean age of patients who had plasma leakage (22 vs 29 years, *p* = 0.007) or severe dengue (19.5 vs. 28 years, *p* = 0.048) was significantly lower compared to patients without these outcomes. The level of education, individual income or having a chronic co-morbidity had no association with an adverse disease outcome (Additional file 1: Table S3). A higher median AST level (73.5 vs. 41 IU/l, *p* = 0009) was associated with plasma leakage but not with severe dengue (Additional file 1: Table S4). None of the other laboratory parameters were statistically significant. Patients with a positive NS1 test were not more likely to have severe dengue (RR: 0.93, 95% CI: 0.55–1.59) or plasma leakage (RR: 1.3, 95% CI: 0.96–1.76) compared to those negative for the test. Similarly, neither the early clinical features nor laboratory investigations had an association with NS1 antigen positivity (data not shown). However, all patients who had spontaneous bleeding (n-7) were NS1 antigen positive though a comparison was not possible as bleeding episodes were not reported in the NS1 negative group.

### Cross-serotype comparison of patients

None of the early clinical or laboratory parameters were distinctly associated with DENV-2 infections compared to non-DENV-2 infections (Additional file 1: Tables S5 and S6). DENV-2 infections were as likely as non-DENV-2 infections to have a positive NS1 test (RR: 1.02, 95% CI: 0.6–1.76). All cases of severe dengue and plasma leakage were reported within DENV-2 group.

## Discussion

In this prospective observational study of clinically suspected dengue patients admitted to the NHSL, plasma leakage (critical phase) and severe dengue were observed in 23.3 and 8.2% of the sample respectively. The first available neutrophil, lymphocyte count, and the median AST level were significantly different in confirmed dengue patients compared to others. LAMP may perform better than commercially available NS1 antigen test in confirming a diagnosis of dengue within the first 3 days of fever. The subgroup of dengue patients with plasma leakage had a significantly lower mean age and a higher median AST level on admission.

### Clinical assessment overestimates the number of dengue patients

Clinical suspicion overestimated the true number of patients by 41.8% (36/122). As these samples were taken early in the course of illness where viremia is highly likely, the results of diagnostic tests are reliable. In addition, none of the 36 unconfirmed patients had a critical phase in their illness and the leukocyte counts and median AST levels were significantly different between the two groups on admission. All dengue cases are reported in the National Surveillance system of Sri Lanka when a “clinically suspected case” of dengue is encountered. A small proportion of these cases will be confirmed eventually by NS1 antigen testing (in early disease) or anti-dengue IgM test (later in the disease) both of which are not readily available in the public health sector. Overall, this may increase the margin of error towards overestimating the number of cases. On the other hand, with our experience as clinicians, at times of epidemics where the health care resources are stretched to the limits, filling paperwork to report cases becomes less of a priority. Hence under-reporting by healthcare staff may partially offset the overestimation of cases by clinical diagnosis.

### RT-LAMP may be a feasible alternative to NS1 testing

Though not as straight forward as NS1 testing, RT-LAMP is cheaper than RT-qPCR. The sensitivity of RT-LAMP against the “gold standard” RT-qPCR in this study was less when compared to previous studies that did a similar comparison for dengue (73.8% vs. 92.5–100%) and comparable in terms of specificity (95.2% vs. 87.5–99.6%) [21, 22]. However, the BLCM showed LAMP to perform as good as RT-qPCR. Latent class models with two latent classes are widely used to estimate the prevalence, sensitivities and specificities in the absence of a gold standard [23]. Comparisons of RT-LAMP vs. NS1 testing is limited in literature and a single study by Sahni et al. from India, who found a sensitivity and specificity of 94.54 and 88.84%% respectively for RT- LAMP vs. NS1 antigen test [21]. In terms of logistics, LAMP is relatively faster to run with 30–45 min hands on time, results can be read with naked eye, requires less capital equipment cost and more amenable to set up in rural hospitals compared to RT-qPCR [24]. However, at this stage neither LAMP nor RT-qPCR can be performed directly on blood and require a RNA extraction step which adds to the cost and time (c.f. NS1 antigen test).

### Younger age and higher AST level at baseline may be useful in predicting plasma leakage

The median baseline AST level was significantly higher, and the mean age was significantly lower in the subgroup which subsequently had plasma leakage. Mean age was also significantly lower in the subgroup that had severe dengue (there was no association with median AST level in this subgroup). Interestingly, the Alanine Aminotransferase (ALT) level (which is more specific to the liver) did not show a significant association. It is plausible that the source of the early rise in AST can either be the liver, muscle or a combination of both. A similar study carried out in the same hospital showed an association of liver tenderness in early infection and plasma leakage. Therefore, presence of early hepatitis in this subgroup cannot be ruled out [25].

Several other studies have previously commented on predictors of severe dengue based on clinical and demographic factors using the same definitions as in this study. A retrospective study in Singapore found that younger age, female gender, duration of fever, having fever on admission, vomiting and abdominal distension to be significant predictors of severe dengue in a logistic regression analysis [26]. While the clinical parameters cannot be compared with our study as ours was a prospective study that correlated early clinical features with subsequent disease outcome, the demographic features can still be compared. While the sex distribution was largely similar between the two studies (male predominated), the Singaporean cohort was on average 10 years older than our cohort and yet the risk of severe dengue decreased with increasing age. However, both these studies did not assess patients at extremes of age who are more vulnerable to complications. A second study in India, where case fatality was also observed, concluded that late presentation to hospital (> 5 days of fever), and dyspnea at rest to be significant predictors of severe disease while age (> 24 years), dulled sensorium and dyspnea at rest were predictors of mortality [27]. Using dyspnea at rest as a predictor of severe dengue encompassing the whole duration of illness is not useful as it can be due to pulmonary edema resulting from iatrogenic fluid overload. In our study we could not detect any relationship between early onset dyspnea and dengue severity. A similar study in Sri Lanka done at a different hospital in the adjoining Gampaha district has shown that both raised AST and ALT levels to be associated with severe dengue in D3 of the illness. These results are more comparable to ours. Another study in China suggests that the ratio between AST/platelet count to be a useful predictor of severe dengue, though this study also was retrospective in nature and data collection was not restricted to early phase of the illness [28]. To have any significant effect on patient management (as prediction of plasma leakage is the goal), it is better to assess early clinical features or laboratory investigations rather than those recorded throughout the entire illness. In this regard, there are not many studies to compare our results with.

### DENV-2 serotype did not cause a distinctly different illness

In this cohort, DENV-2 predominated over other serotypes. A recently completed meta-analysis concluded that on a global scale, the highest pooled mortality rate was observed in DENV-2 dominated epidemics compared to others [29]. This may be due to an intrinsically higher virulence of this serotype. However, we could not find any evidence for a distinct illness phenotype caused by DENV-2. This observation fits well with the hypothesis that a change in the severity of an epidemic is linked to a shift between two serotypes (with a non-immune population being exposed to a different serotype) rather than due to an intrinsic virulence of a particular serotype. All severe cases of dengue and all cases with plasma leakage in this sample belonged to DENV-2 serotype but given the small number of samples from non-DENV2 serotypes, it is premature to attribute it as a trait of the serotype.

### Future directions

Unlike in many previous studies, this cohort was a prospective cohort that correlated clinical or laboratory investigation values obtained in early infection with subsequent disease outcomes with the best chance of picking parameters with real predictive value. However, the associations found need to be further strengthened for sensitivity and specificity by incorporating more parameters with predictive value by more advanced analysis of the virus (phylogenetics, heritability, epistasis) and host response (immunogenetics, host cytokine profile). Better collaborations between clinicians and basic scientists would complement each other’s expertise in making more accurate, cost-effective risk prediction algorithms that can be implemented at bedside in real-time.

### Limitations

The circulating dengue serotype was predominantly DENV-2 during the sample collection for this study which prevented a detailed comparison with each of the other serotypes. The reporting of first day of fever is subject to recall bias. This study was restricted to one teaching hospital in the country. However, this hospital is in the most populous district of the country where the largest number of dengue cases has been reported annually for the last 5 years. We do not expect the patient profile reported in this hospital to be different from any other hospital in the country though patients at NHSL may have better outcomes than those managed at the periphery due to better facilities (e.g. access to ultrasound scans). Finally, the number of patients within severe dengue category was small and this could have obscured any significant findings due to inadequate power.

## Conclusion

In this prospective study of clinically suspected dengue patients in Sri Lanka, 3 out 10 patients were not confirmed with a diagnostic test. The confirmed dengue patients had significantly higher AST levels and lower lymphocyte counts in early disease compared to others. LAMP outperformed NS1 antigen test for diagnosis and was as good as RT-qPCR when compared in a model that assumes no “gold standard”. In confirmed dengue patients, younger age and a higher AST level in early infection were associated with subsequent plasma leakage. Validation of the use of LAMP for dengue diagnosis with a validation cohort in future can be useful.

## Additional files

Additional file 1: **Table S1.** Clinical signs and symptoms of enrolled patients. **Table S2.** Laboratory parameters of enrolled patients. **Table S3.** Socio-demographic features and dengue severity. **Table S4.** Associations between laboratory investigations within the first 3 days of fever and the adverse outcomes in dengue. **Table S5.** Comparison of clinical features of patients infected with DENV-2 and others. **Table S6.** Comparison of laboratory investigations (within the first 3 days of fever) between patients infected with DENV-2 and others (median (Q1-Q3)). (DOCX 44 kb)

Additional file 2: Methods. (DOCX 167 kb)

## Data Availability

The datasets used and analyzed during the current study are available from the corresponding author on request.

## Abbreviations

ALT: Alanine Aminotransferase
AST: Aspartate Aminotransferase
BLCM: Bayesian Latent Class Model
BMI: Body Mass Index
CDS: Colombo Dengue Study
LAMP: Loop-mediated isothermal amplification
NHSL: National Hospital of Sri Lanka
NPV: Negative Predictive Value
NS1: Non-Structural protein 1
PPV: Positive Predictive Value
RT-qPCR: Reverse Transcription and quantitative Polymerase Chain Reaction
WHO: World Health Organization
