# Supplementary material for: Risk prediction for severe disease and better diagnostic accuracy in early dengue infection; the Colombo dengue study
Source: BMC Infect Dis. 2019 Aug 1;19:680. doi: 10.1186/s12879-019-4304-9 (PMC6676631; doi:10.1186/s12879-019-4304-9)
Supplement: Supplementary file 1 — Table S1. Clinical signs and symptoms of enrolled patients. Table S2. Laboratory parameters of enrolled patients. Table S3. Socio-demographic features and dengue severity. Table S4. Associations between laboratory investigations within the first 3 days of fever and the adverse outcomes in dengue. Table S5. Comparison of clinical features of patients infected with DENV-2 and others. Table S6. Comparison of laboratory investigations (within the first 3 days of fever) between patients infected with DENV-2 and others (median (Q1-Q3)). (DOCX 44 kb) [file 12879_2019_4304_MOESM1_ESM.docx]

**Additional file 1**

**Table S1** Clinical signs and symptoms of enrolled patients

| ***Clinical feature*** | ***Confirmed dengue patients (n-86)*** | | ***Treated as dengue fever without confirmation (n-36)*** | |
| --- | --- | --- | --- | --- |
|  | ***Number*** | ***%*** | ***Number*** | ***%*** |
| Headache | 70 | 81.40 | 22 | 61.11 |
| Myalgia | 65 | 75.58 | 22 | 61.11 |
| Arthralgia | 47 | 54.65 | 17 | 47.22 |
| Diarrhea | 18 | 20.93 | 5 | 13.89 |
| Abdominal pain | 27 | 31.40 | 7 | 19.44 |
| Dyspnea | 3 | 3.49 | 1 | 2.78 |
| Vomiting | 17 | 19.77 | 11 | 30.56 |
| Retro-orbital pain | 13 | 15.12 | 4 | 11.11 |
| Bleeding | 7 | 8.14 | 2 | 5.56 |
| Hepatomegaly | 2 | 2.35 | 0 | 0 |

**Table S2** Laboratory parameters of enrolled patients

| ***Laboratory parameter*** | ***Confirmed dengue patients (n-86)*** | | | ***Treated as dengue fever without confirmation (n-36)*** | | |
| --- | --- | --- | --- | --- | --- | --- |
|  | ***Median value*** | ***Number of patients*** | ***Median day of illness*** | ***Median value*** | ***Number of patients*** | ***Median day of illness*** |
| Haemoglobin (g/dL) | 13.65 | 86 | 2 | 11.35 | 36 | 2 |
| Haematocrit (%) | 39.45 | 86 | 2 | 33.80 | 36 | 2 |
| Platelet count 10^3^/uL | 126 | 86 |  | 147 | 36 | 2 |
| Total leukocyte count* | 4.12 | 86 | 2 | 6.54 | 36 | 2 |
| Neurophil count * | 2.39 | 86 | 2 | 3.78 | 36 | 2 |
| Lymphocyte count * | 0.70 | 86 | 2 | 1.19 | 36 | 2 |
| AST^#^ * | 50.0 | 86 | 2 | 27.0 | 36 | 2 |
| ALT^##^ | 35.5 | 86 | 2 | 25.5 | 36 | 2 |
| Serum Sodium | 136 | 85 | 3 | 134 | 36 | 2 |
| Serum Potassium | 3.7 | 85 | 3 | 3.8 | 36 | 2 |
| Serum creatinine | 78.0 | 85 | 3 | 73.5 | 36 | 2 |
| C-reactive protein | 16.0 | 85 | 2 | 06.0 | 35 | 2 |
| Serum total bilirubin | 11.6 | 47 | 3 | 12.8 | 19 | 2 |

Normal distribution of continuous variables were tested by Shapiro-wilk test. BMI was distributed normality. BMI was analysed between confirmed and non-confirmed dengue patients using T test. Other laboratory parameters were analysed by Mann-Whitney test, ^#^Alanine aminotransferase, ^##^Aspartate aminotransferase

* Statistically significant with the Bonferroni adjusted p value of <0.004

**Table S3** Socio-demographic features and dengue severity

|  | ***Plasma leakage***  ***(n-86)*** | | ***RR (95% CI)*** | ***Severe dengue***  ***(n-86)*** | | ***RR (95% CI)*** |
| --- | --- | --- | --- | --- | --- | --- |
|  | Yes  (n-22) | No  (n-64) |  | Yes  (n-10) | No  (n-76) |  |
| Age (median) | 22** | 29** | - | 19.5* | 28* | - |
| BMI (median) | 20.73 | 21.04 | - | 20.35 | 21.04 | - |
| *Gender*  Female  Male | 6  16 | 23  41 | 0.74 (032-1.68) | 2  8 | 27  49 | 0.49 (0.11-2.17) |
| *Level of education*  Passed GCE O/L or above  GCE O/L not complete  *Level of monthly income of individuals*  Above national average income per capita  Below national average income per capita  *Preexisting co-morbidity*  Hypertension  Yes  No  Hyperlipidemia  Yes  No  Diabetic Mellitus  Yes  No  Presence either Hypertension, Hyperlipidemia, IHD and Diabetic Mellitus  Yes  No  IHD  Yes  No  Smoking  Yes  No  Alcohol consumption  Yes  No | 17  5  14  8  2  20  2  20  0  22  2  20  0  22  3  19  3  19 | 45  19  46  18  6  58  2  62  4  60  11  53  3  61  12  52  7  57 | 1.32 (0.55-3.17)  0.76 (0.36-1.58)  0.98 (0.28-3.43)  2.05 (0.72-5.87)  0  0.56 (0.15-2.12)  0  0.75 (0.25-2.21)  1.2 (0.43-3.34) | 10  0  6  4  1  9  1  9  0  10  1  9  0  10  2  8  1  9 | 52  24  54  22  7  69  3  73  4  72  12  64  3  73  13  63  9  67 | -  0.65 (0.2-2.11)  1.08 (0.16-7.49)  2.28 (0.37-13.86)  0  0.62 (0.09-4.52)  0  1.18 (0.28-5.02)  0.84 (0.12-5.98) |

* p<0.05, ** p<0.01

**Table S4** Associations between laboratory investigations within the first 3 days of fever and the adverse outcomes in dengue

| ***Clinical features of confirmed dengue patients*** | ***Presence/ absence*** | ***Haemoglobin (median)*** | ***Haematocrit (mean)*** | ***Total leukocyte count (median)*** | ***Platelets (median)*** | ***Neutrophils (median)*** | ***Lympocytes (median)*** | ***AST (median)*** | ***ALT (median)*** | ***Serum Sodium (median)*** | ***Serum Potassium (median)*** | ***Serum creatinine (median)*** | ***C-reactive protein (median)*** | ***Serum total bilirubin (median)*** |
| --- | --- | --- | --- | --- | --- | --- | --- | --- | --- | --- | --- | --- | --- | --- |
| Severe dengue (n-86) | Yes  (n-10) | 14.75 | 40.93 | 4.46 | 98.5 | 3.15 | 0.71 | 73 | 40 | 135.5 | 3.75 | 77.5 | 23 | 12.55 |
|  | No  (n-76) | 13.6 | 38.58 | 4.07 | 128 | 2.36 | 0.70 | 47.5 | 35.5 | 136 | 3.70 | 78 | 14.1 | 10.3 |
| Plasma leakage  (n- 86) | Yes  (n-22) | 14.75 | 40.64 | 3.76 | 95 | 2.44 | 0.70 | 73.5** | 51 | 135.5 | 3.7 | 78 | 23 | 11.7 |
|  | No  (n-64) | 13.50 | 38.24 | 4.12 | 137 | 2.39 | 0.72 | 41** | 35 | 136 | 3.7 | 77 | 8.6 | 10.75 |

** Statistically significant with the Bonferroni adjusted p value of <0.004

**Table S5** Comparison of clinical features of patients infected with DENV-2 and others

| ***Clinical features of dengue patients*** | | ***DENV 2***  ***(n-70)*** | ***Non DENV-2***  ***(n-10)*** | ***RR (95% CI)*** |
| --- | --- | --- | --- | --- |
| Headache | Yes | 57 | 8 | 1.01 (0.81-1.26) |
|  | No | 13 | 2 |  |
| Myalgia | Yes | 55 | 6 | 1.14 (0.89-1.46) |
|  | No | 15 | 4 |  |
| Arthralgia | Yes | 38 | 5 | 1.02 (0.86-1.20) |
|  | No | 32 | 5 |  |
| Diarrhea | Yes | 13 | 3 | 0.91 (0.71-1.17) |
|  | No | 57 | 7 |  |
| Abdominal pain | Yes | 21 | 4 | 0.94 (0.78-1.15) |
|  | No | 49 | 6 |  |
| Dyspnea | Yes | 3 | 0 | 1.15 (1.05-1.25) |
|  | No | 67 | 10 |  |
| Vomiting | Yes | 16 | 1 | 1.10 (0.94-1.28) |
|  | No | 54 | 9 |  |
| Retro-orbital pain | Yes | 13 | 0 | 1.18 (1.06-1.30) |
|  | No | 57 | 10 |  |
| Bleeding | Yes | 5 | 1 | 0.95 (0.66-1.37) |
|  | No | 65 | 9 |  |
| Hepatomegaly | Yes | 1 | 0 | 1.14 (1.05-1.25) |
|  | No | 69 | 10 |  |

* Statistically significant with the Bonferroni adjusted p value of <0.005

**Table S6** Comparison of laboratory investigations (within the first 3 days of fever) between patients infected with DENV-2 and others (median (Q1-Q3))

| ***Laboratory parameter*** | ***DENV 2***  ***(n-70)*** | ***Non-DENV 2***  ***(n-10)*** | |
| --- | --- | --- | --- |
| Haemoglobin (g/dL) | 13.70 (11.70-14.70) | 11.95 (11.58-14.83) | |
| Haematocrit (%) | 39.45 (34.48-42.83) | 35.65 (34.68-42.03) | |
| Platelet count 10^3^/Ul | 130 (87.50-173.00) | 151 (124.50-207.75) | |
| Total leukocyte count | 3.88 (2.81-5.32) | 4.80 (3.45-9.14) | |
| Neutrophil count | 2.32 (1.51-3.72) | 3.69 (2.14-7.43) |  |
| Lymphocyte count | 0.70 (0.48-1.10) | 0.69 (0.48-1.05) |  |
| AST | 51 (29.50-79.50) | 38.5 (28.25-57.00) |  |
| ALT | 35 (22.00-65.75) | 45 (19.75-65.00) |  |
| Serum Sodium | 136 (134-137) | 136 (135-138) |  |
| Serum Potassium | 3.60 (3.25-4.10) | 3.85 (3.40-4.13) |  |
| Serum creatinine | 78.00 (67.50-97.00) | 78.5 (68.75-90.00) |  |
| C-reactive protein | 14.20 (6.00-33.15) | 38.5 (6.75-58.38) |  |
| Serum total bilirubin | 11.05 (8.68-15.95) | 11.95 (8.65-20.48) |  |

* Statistically significant with the Bonferroni adjusted p value of <0.004
