# Supplementary material for: Risk prediction for severe disease and better diagnostic accuracy in early dengue infection; the Colombo dengue study
Source: BMC Infect Dis. 2019 Aug 1;19:680. doi: 10.1186/s12879-019-4304-9 (PMC6676631; doi:10.1186/s12879-019-4304-9)
Supplement: Supplementary file 2 — Methods. (DOCX 167 kb) [file 12879_2019_4304_MOESM2_ESM.docx]

**Additional file methods**

*NS 1 antigen testing*

Samples were tested for NS1 antigen detection using commercially available one step SD Bioline dengue NS 1 antigen test manufactured by Alere SD (USA) following the manufacturer’s recommendations and classified them as NS1 positive and negative according to the manufacturer’s instructions.

*qRT-PCR test and serotyping*

The DENV multiplex qRT-PCR was performed using the SuperScript III Platinum One-Step qRT-PCR kit. Reaction mixtures were scaled from the manufacturer-recommended 50 mL volume to 25 mL per reaction. Each reaction contained 300 nM primers for DENV-1, -2, and -3 and 450 nM primers for DENV-4. Each probe was added to 600 nM. In the final PCR reaction, and 5 mL of RNA eluate was added to each reaction. qRT-PCR reactions were performed using the Rotor-Gene Q instrument. Cycling conditions were the following: 52uC for 15 min (RT step); 94uC for 2 min; 45 cycles of 94uC for 15 sec, 55uC for 20 sec, 60uC for 20 sec, and 68uC for 20 sec (run time, 136 min). Detection was performed in the green, yellow, orange, and red channels at 55uC; the gain was set at 10 for green, yellow, and orange, and at 5.33 for red. Four-step cycling was initially used to detect signal at different temperatures, but it was maintained as it showed improved sensitivity and curve generation compared to standard, three-step cycling. During analysis, slope correction was performed for each channel. Additionally, the first five cycles were cropped from the orange and red channel to improve baseline normalization. The threshold was set at 0.05 for green and yellow and 0.025 for orange and red. A positive result was considered any curve crossing this threshold prior to cycle 40. Serotype was determined based on the pattern of signals obtained from the four DENV probe. Ct value of 38.5 was used as the cut off value for RT-PCR test .

qRT-LAMP testing

The genomic viral RNA will be extracted from 140ul of patient plasma samples using QIAamp RNA mini kit (Qiagen, Germany) at the Department of Parasitology, Faculty of Medicine, University of Colombo, Sri Lanka. The RNA will be eluted in a final volume of 60 ul of elution buffer.

Primers for the detection dengue virus and the loop primers used for the RT-LAMP assay was synthesized. The RT-LAMP assay was performed using Loopamp RNA amplification kit (Eiken Chemical Co. Ltd, Japan). Briefly, a 25ul reaction mixture consisted of 1.6 uM FIP and BIP primer, 0.8 uM Loop F and Loop B primer, 0.2 uM F3 and B3 primer, 12.5 ul of 2x reaction mixture (RM), 1ul of enzyme mixture (EM), 0.7 ul of sterile deionized water, 1ul of Florescent detection reagent (FD) and 1ul of template RNA.


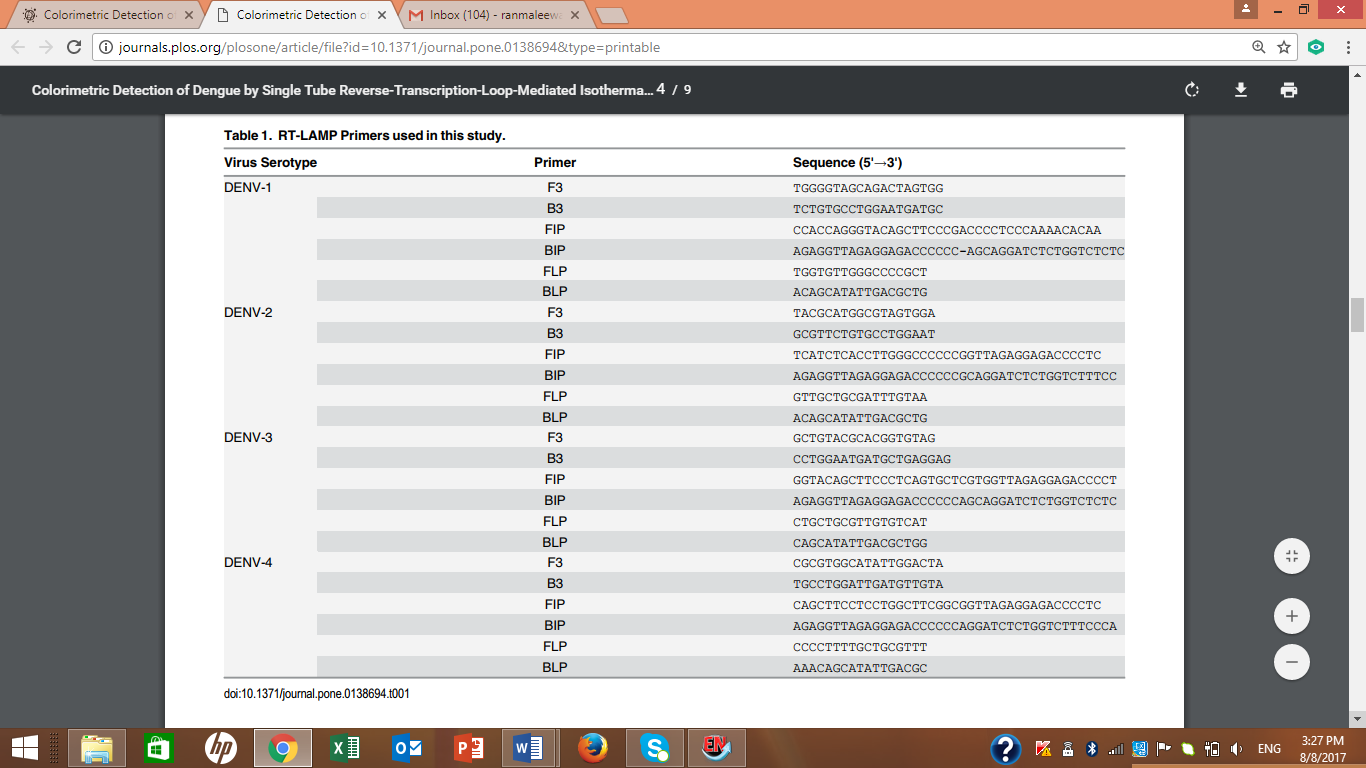
Figure *1:*  Primers used for *RT-LAMP testing* ^1^

Single tube RT-LAMP was conducted by adding all primer sets in a single reaction mixture. The reaction mixture was carried out in Loopamp real time turbidimeter. All experiments was performed in duplicates and as explained by the study in Malaysia, carried out in 2015. HNB dye (Sigma, USA) was dissolved in distilled water at 20mM to prepare a stock solution. The RT-LAMP assay containing 120uM of HNB dye was performed in a 25ul reaction mixture with the same components as in the RT-LAMP assay except excluding the FD reagent. Turbidity of the RT-LAMP assay was observed with the naked eye. A positive reaction was indicated by colour change from violet to sky blue.

References

1. Lau Y-L, Lai M-Y, Teoh B-T, et al.; Colorimetric Detection of Dengue by Single Tube Reverse-Transcription-Loop-Mediated Isothermal Amplification. *PLOS ONE* 2015;**10**(9):e0138694. doi: 10.1371/journal.pone.0138694.
